# Enzymatic Metabolism of Vitamin A in Developing Vertebrate Embryos

**DOI:** 10.3390/nu8120812

**Published:** 2016-12-15

**Authors:** Melissa A. Metzler, Lisa L. Sandell

**Affiliations:** Department of Molecular, Cellular and Craniofacial Biology, University of Louisville, Louisville, KY 40201, USA; Melissa.Metzler@Louisville.edu

**Keywords:** retinoic acid, review, metabolism, signaling, embryo, development, Rdh10, Dhrs3, retinoid, vitamin A

## Abstract

Embryonic development is orchestrated by a small number of signaling pathways, one of which is the retinoic acid (RA) signaling pathway. Vitamin A is essential for vertebrate embryonic development because it is the molecular precursor of the essential signaling molecule RA. The level and distribution of RA signaling within a developing embryo must be tightly regulated; too much, or too little, or abnormal distribution, all disrupt embryonic development. Precise regulation of RA signaling during embryogenesis is achieved by proteins involved in vitamin A metabolism, retinoid transport, nuclear signaling, and RA catabolism. The reversible first step in conversion of the precursor vitamin A to the active retinoid RA is mediated by retinol dehydrogenase 10 (RDH10) and dehydrogenase/reductase (SDR family) member 3 (DHRS3), two related membrane-bound proteins that functionally activate each other to mediate the interconversion of retinol and retinal. Alcohol dehydrogenase (ADH) enzymes do not contribute to RA production under normal conditions during embryogenesis. Genes involved in vitamin A metabolism and RA catabolism are expressed in tissue-specific patterns and are subject to feedback regulation. Mutations in genes encoding these proteins disrupt morphogenesis of many systems in a developing embryo. Together these observations demonstrate the importance of vitamin A metabolism in regulating RA signaling during embryonic development in vertebrates.

## 1. Introduction

During development an embryo must build itself from a single cell into a complex organism, a process that requires cells and tissues to communicate with each other to organize an overall body plan and morphogenesis of organs and structures. The cell interactions essential for development are mediated by a small number of molecular signaling pathways, including wnt, hedgehog (HH), fibroblast growth factor (FGF), transforming growth factor (TGF), and retinoic acid (RA) signaling. Vitamin A is essential for embryogenesis because it is the molecular precursor of the signaling molecule RA. This review will describe the metabolism of vitamin A to RA in developing embryo tissues, highlighting reversible reaction steps that occur in association with membranes, and will provide a brief overview of aspects of embryo development that depend upon RA signaling. (NOTE: Throughout the review we will follow nomenclature rules for mouse when collectively referring to genes or proteins from multiple species, and will use appropriate nomenclature rules when referring to genes or proteins from specific organisms.)

Although the molecular identity of vitamin A was not identified until the twentieth century, the value of the nutrient to human health has been appreciated since ancient times. The function, identity, and molecular nature of vitamin A were elucidated cumulatively over the course of many years and many studies (reviewed in [1,2]). The requirement for vitamin A in embryonic development was first addressed experimentally by nutritional experiments performed in the 1930s and 1940s. Newborn pigs birthed from gilts fed diets deficient in vitamin A were born without eyes, demonstrating that vitamin A is needed for eye morphogenesis [3,4,5]. Fetal and newborn rats from mothers fed vitamin A deficient diets had severe malformations. The spectrum of vitamin A deficiency defects included abnormalities in development of the eye, face, palate, heart, limb, genito-urinary tract, diaphragm, lung, salivary glands, intestinal villi, skeleton, and nasal region [6,7,8,9,10,11,12,13,14]. The array of malformations resulting from such diets became known collectively as vitamin A deficiency syndrome. Studies of avian embryos elucidated molecular and cellular mechanisms underlying defects resulting from vitamin A deficiency [15,16,17,18,19].

Developmental defects occur not only under conditions of deficiency. Exposure to excess vitamin A or its active form, RA, is equally deleterious for embryonic development as is vitamin A deficiency [20,21,22,23,24,25,26,27]. Thus, too little or too much vitamin A, or its active derivative RA, disrupts embryonic development.

## 2. Vitamin A is the Molecular Precursor of RA

**Figure 1:**
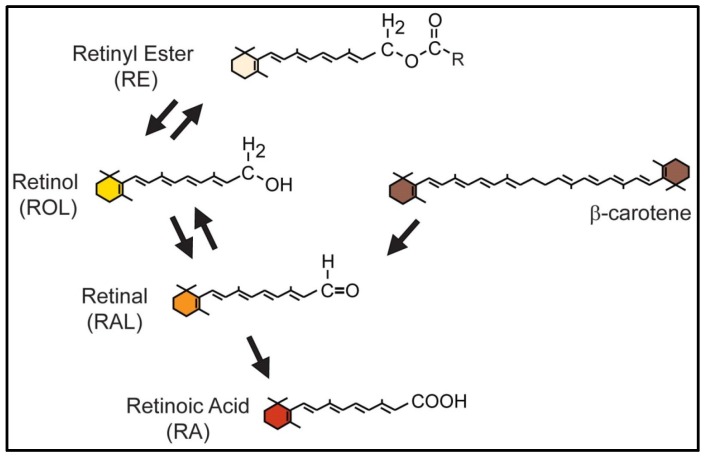
Retinoid compounds differ with respect to the polar end group at the terminus of the chain. The compound all-*trans*-retinol (ROL) can be reversibly converted to all-*trans*-retinal (RAL) or to retinyl esters (RE). The carotenoid β-carotene can be converted to RAL. RAL can be irreversibly oxidized to retinoic acid (RA).

The chemical structure of vitamin A was solved by Karrer and colleagues in 1931 [28]. Strictly applied, the term “vitamin A” describes the molecule all-*trans*-retinol (ROL) [29]. More loosely applied, the term can be used to refer to set of compounds closely related to ROL. Collectively ROL and its derivatives are known as “retinoids”, a term that can be applied to natural or synthetic molecules with vitamin A activity. In this review, the term retinoid refers to natural compounds only. Each retinoid is composed of a monocyclic ring with an attached isoprenoid chain (Figure 1). The individual retinoids differ with respect to the polar end group at the terminus of the chain (reviewed in [30]). ROL, the alcohol form, has a terminal –OH hydroxyl group. Retinyl esters (RE) have a terminal –O-alkyl unit. The aldehyde form of vitamin A, all-*trans*-retinal (RAL), has a terminal –CHO aldehyde group. RA, the acid form of vitamin A has a –COOH in the terminal position. ROL and RAL are critical for energy metabolism [31,32] and vision (reviewed in [33]). With respect to embryo development, RA is the retinoid that mediates cellular signaling and transcriptional regulation critical for pattern formation and organogenesis. RA has the most potency in rescuing the developmental defects associated with vitamin A deficiency [34]. Inversely, in excess, RA is the most potent teratogen [35]. 

The relationship and interconversion of the retinoid forms was described initially by Dowling and Wald [36]. ROL can be reversibly converted to RE, or to RAL (Figure 1). Additionally, RAL can be generated by cleavage of carotenoids such as β–carotene. RAL can then be oxidized to form RA [37]. The oxidation of RAL to RA is a non-reversible reaction. All retinoids are polarized and highly hydrophobic [38]. ROL localizes to membranes and lipid compartments within cells and tissues [39]. In environments such as cytosol or blood serum, retinoids are bound by proteins protecting them from aqueous surroundings.

Animals cannot synthesize any retinoids de novo but must obtain the molecules as micronutrients from the diet. Vitamin A can be taken up in two forms; “preformed vitamin A”, which refers to the retinoid molecules; or “provitamin A”, which refers to carotenoids (reviewed in [40,41]). Preformed vitamin A, predominantly in the form of ROL or RE, can be obtained from animal sources such as milk, eggs, and liver. Provitamin A carotenoids, including β-carotene, are obtained from plant sources such as sweet potato and carrots.

Embryos receive retinoids from their mother. In mammals, maternal-embryo retinoid transfer occurs via transmission of ROL or β-carotene across the placental barrier [42,43,44]. In egg-laying species, maternal-embryo retinoid transfer occurs by maternal deposition of retinoids and carotenoids to the egg yolk [45,46], and subsequent embryo uptake from the yolk. The form of retinoid stored in egg varies in different evolutionary groups. Fish eggs have more RAL than ROL, and birds and reptiles have more ROL than RAL. Once the cells of an embryo take up ROL or β-carotene, these precursors can be oxidized to RAL, and then to the active signaling molecule RA.

## 3. RA Signaling Occurs via RAR Nuclear Transcription Factors

**Figure 2:**
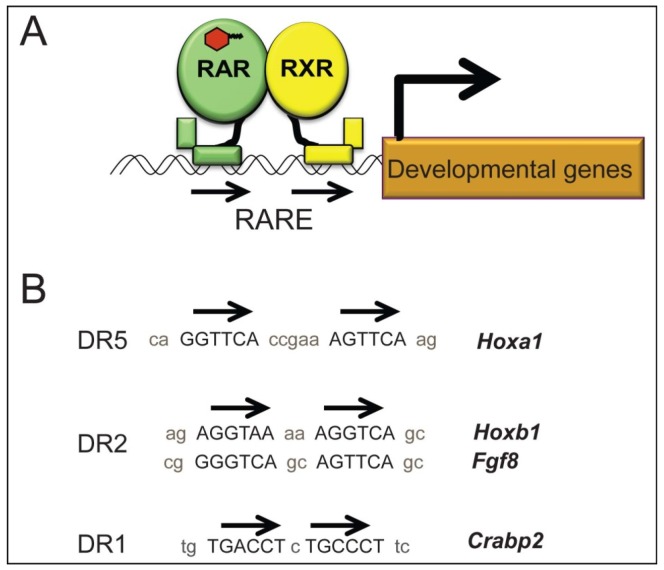
Canonical RA signaling activity occurs when RA regulates gene transcription by binding as ligand to RAR nuclear transcription factors at RARE sites. (**A**) RARs function as heterodimer with RXRs. Binding of RA (represented by orange hexagon) to the ligand-binding domain of a RAR heterodimerized with a RXR can activate transcription; (**B**) RAR–RXR heterodimers bind to DNA at RARE sites. Examples of classical RARE binding sites consist of two directly repeated hexamer motifs separated by spacer nucleotide sequences of different lengths. Examples of DR5, DR2, and DR1 RAREs from important RA-regulated developmental genes are shown.

The most fully elucidated mechanism of RA signaling is the canonical pathway in which RA regulates transcription by binding to specific nuclear transcription factors known as retinoic acid receptors (RARs) (Figure 2A) (reviewed in [47,48,49,50]). RARs function as heterodimers with related transcription factors retinoid X receptors (RXRs). There are three RAR subtypes, (RARα, RARβ, RARγ). Most vertebrates have three RAR genes, one for each receptor subtype. Zebrafish have two RAR alpha and two RAR gamma but no RAR beta [51]. There are typically three RXRs (RXRα, RXRβ, RXRγ), although fishes generally have one additional RXR, and the zebrafish, in particular, have duplicates of each RXR gene [52,53]. Some of the *Rar* and *Rxr* genes are expressed in specific tissues of developing embryos, while others are broadly expressed [54]. Multiple RAR/RXR heterodimeric combinations can transduce RA signaling. Mouse embryos exposed to excess RA have active signaling in nearly all cells of the embryo as detected by a transgenic reporter [55]. Ectopic expression of RAR does not result in ectopic signaling, demonstrating that RAR expression is not limiting [56]. Thus, within a given embryonic cell or tissue, the presence or absence of RA signaling is defined primarily by presence or absence of RA, and not by the expression of RARs.

In addition to their role as partners for RARs, RXRs can interact functionally with other nuclear receptors including thyroid hormone receptors, vitamin D receptor, peroxisome proliferator-activated receptors (PPARs), and others [57,58]. With respect to RA signaling, it is particularly notable that RXRs interact with PPARβ/δ, which can bind RA and transduce signal [59,60,61]. RA signaling through RXR:PPARβ/δ has been demonstrated to be important for energy homeostasis and insulin response [62], but a role for this combination of receptors in embryonic development has not been defined.

RAR/RXR heterodimers can bind constitutively to target DNA sequences known as RA response elements (RAREs) located in the enhancer regions of genes regulated by RA (Figure 2A). Typically RAREs consist of two hexamer binding “half-sites”, arranged as direct repeats (DR), inverted repeats (IR) or palindromes (Figure 2B) [63,64,65]. The half-sites may be separated by 0–10 nucleotides. The different configurations are denoted DR0, DR1, DR2, IR9, IR10, etc., depending on the relative orientation and the number of non-binding spacer nucleotides between the hexamer binding sequences. Examples of RAREs that regulate expression of genes important for embryonic development include a DR5 that is critical for controlling expression of *Hoxa1* in mouse [66,67], DR2s that regulate expression of *Hoxb1* [68] and *Fgf8* [69], and a DR1 that regulates expression of *Crabp2* [70]. 

The canonical model of RA signaling is that RAR/RXR heterodimers are bound to target DNA elements independent of ligand, and that RA binding activates gene transcription by releasing co-repressors and recruiting co-activating factors [71]. However, recent studies reveal that ligand-dependent activation of constitutively bound receptors is only one of many mechanisms of retinoid signaling. The canonical mode of RARs binding DNA constitutively is not universal. In some cases, RA ligand can induce RAR/RXR binding to unoccupied RAREs in cultured embryonic stem cells [72,73]. Moreover, the canonical mechanism wherein ligand binding activates gene expression is not the only mechanism of RA-mediated gene regulation. In some cases, binding of RA to RARs can cause gene repression rather than activation ([65], reviewed in [74]). RA regulation of gene expression is also not limited to interaction with RARs. RA also functions as a ligand for other nuclear receptors [59,64]. Finally, RA and RAR have alternate functions outside the nucleus unrelated to direct regulation of transcription [58,75].

The role of RAR and RXR receptors in mediating RA signaling during embryogenesis has been elucidated in part by examining the phenotype of genetic loss of function. Mutation of *Rxra* causes defects in formation of the eyes and heart outflow tract, phenotypes also observed in vitamin A deficiency. For the other two *Rxr* genes, and all three *Rar* genes, mutant loss of function does not cause strong embryo phenotypes when mutations are present individually. However, compound mutations of different *Rar* genes exhibit multiple defects. For example, compound mutation of *Rara* and *Rarb*, *Rara* and *Rarg*, or *Rxra* and *Rxrb* each cause a spectrum of embryonic phenotypes similar to that observed in vitamin A nutritional deficiency [50,76]. The lack of strong phenotype in most individual mutants of *Rar* and *Rxr* genes presumably reflects the functional redundancy of these factors.

## 4. Regulation of RA Signaling by Metabolic Enzymes

**Figure 3:**
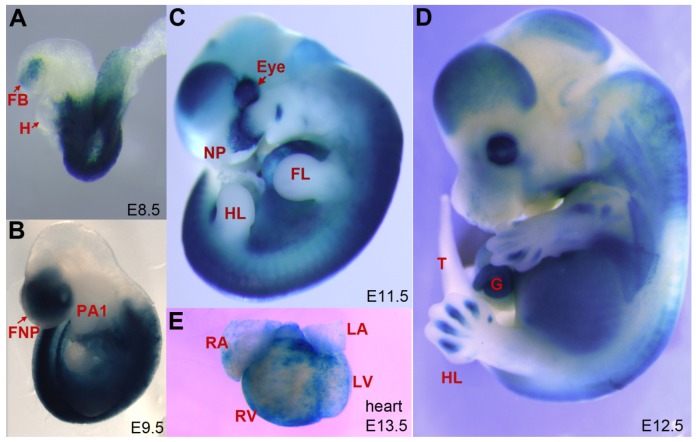
RA is not present uniformly throughout an embryo, and RA signaling activity does not occur equally in all cells. The distribution of canonical ligand-dependent RA signaling in mouse embryos can be visualized by activity of a RARE-lacZ transgenic reporter [55]. (**A**) At embryonic day 8.5 (E8.5) RA signaling occurs predominantly in the trunk and the forebrain. RA negative tissues include the heart and most of head; (**B**) At E9.5 RA signaling remains strong in trunk. RA negative tissues include pharyngeal arch 1, which gives rise to the maxilla and mandible and most of the midbrain and hindbrain; (**C**) At E11.5 strong RA signaling activity is present in the eye, in the developing nasal region and throughout the developing spinal cord of the trunk. Forelimbs and hindlimbs are negative for RA signaling at this stage; (**D**) At E12.5 RA signaling is detected in the eye, in distinct regions of the trunk, and in interdigital regions of the forelimb and hindlimb. The gut, which is outside the body wall at this stage, is strongly positive for RA signal. RA-negative tissues include the tail and digits of the limb; (**E**) RA signaling occurs in many developing organs including the E13.5 heart. E, embryonic day; FB, forebrain; FL, forelimb; FNP, frontonasal prominence; G, gut; H, heart; HL, hindlimb; LA, left atrium; LV, left ventricle; NP, nasal prominence; PA1, pharyngeal arch 1; RA right atrium; RV, right ventricle; T, tail.

RA is not present uniformly throughout an embryo, and RA signaling activity does not occur equally in all cells (Figure 3). Differential distribution of RA in developing embryonic tissues of chick has been detected by direct chemical analysis [77,78]. In zebrafish differential distribution of RA has been visualized by molecular reporters that fluoresce in presence of RA [79]. Spatial distribution of RA signaling activity can also be visualized indirectly by staining for activity of transgenic reporters of canonical ligand-dependent RAR gene activation in mice and zebrafish [55,56,80].

For most key embryonic signaling pathways (Wnt, HH, FGF, TGF, etc.) the distribution of a signal within an embryo reflects the distribution of secreted protein ligand and cognate receptor. Thus, signaling by these pathways is regulated by production, secretion, and post-translational modification of proteins. In contrast, for the RA pathway, the precisely regulated level and distribution of signal activity is controlled by metabolic conversion of inactive retinoids to active RA, and by elimination of RA. Thus, the enzymes and binding proteins that mediate retinoid transport, metabolism, and catabolism are critical regulators of embryonic development.

## 5. ROL Enters Embryo Cells by Transport and Diffusion

**Figure 4:**
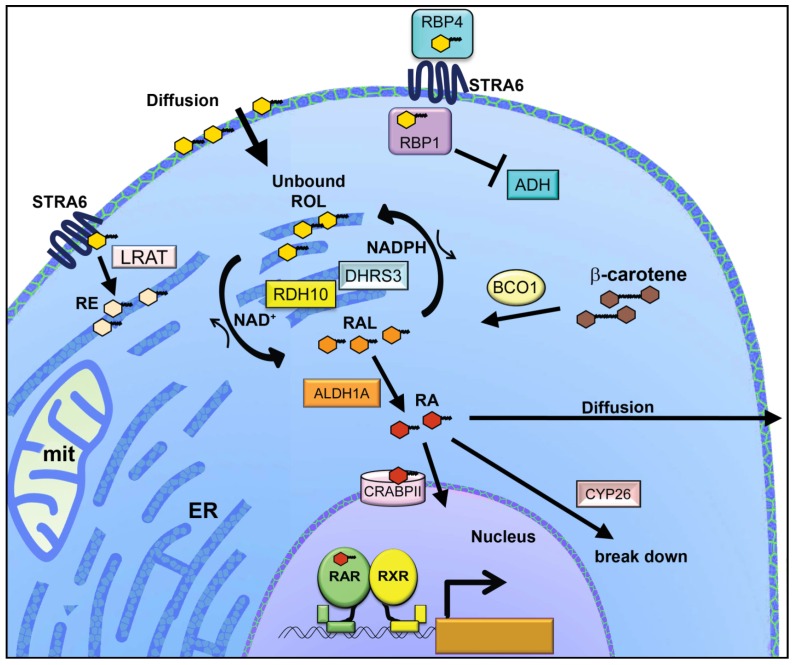
RA production in embryo cells is regulated by enzymatic metabolism and catabolism. Embryo cells obtain retinoids from maternal sources via circulation. In the blood serum the hydrophobic ROL is bound by RBP4. ROL enters embryo cells predominantly through membrane diffusion. ROL can also be transported into cells via RBP4 delivery to the membrane transporter STRA6. Inside the cell hydrophobic ROL can be associated with a membrane compartment, where it can exist as free retinoid. Hydrophobic ROL can be present in the aqueous cytosol if it is bound by RBP1. ADH enzymes do not participate in metabolism of ROL in embryo tissues under normal conditions because the cytoplasmic enzymes cannot act on RBP1-bound ROL. RDH10, a membrane bound SDR, acts on free ROL, possibly from a membrane pool, or alternatively, delivered from RBP1. A related membrane SDR, DHRS3 catalyzes the reduction of RAL to ROL. RDH10 and DHRS3 are oxidoreductases that act reciprocally and enhance the activity of each other. RDH10 functions primarily as an oxidase and DHRS3 acts chiefly as a reductase, the redox directions being defined by relative abundance of cofactors NAD^+^ and NADPH. An alternate mechanism to generate RAL is cleavage of β-carotene by BCO1. RAL is irreversibly converted to RA by members of the ALDH1A family. Once RA is generated by oxidation of RAL, it can have several fates. RA can bind to CRABP2, which can deliver the RA into the nucleus where it can bind to RARs. RA can also be eliminated by action of CYP26 family enzymes, or it can move to neighboring cells, possibly by diffusion or transport. ER, endoplasmic reticulum; mit, mitochondrion.

Cells of a developing embryo receive retinoid from the placenta or egg yolk via the embryonic circulation. Within the blood serum ROL is bound by retinol binding protein 4 (RBP4) ([81] reviewed in [82,83]). ROL can enter the cells of an embryo by two methods; transport through a membrane transporter, or membrane diffusion (Figure 4). For the transport mechanism, RBP4 delivers ROL to the membrane transporter STRA6, which transfers the ROL into the cell and delivers it to the cellular retinol binding protein RBP1 (also known as CRBP1) [84,85,86], reviewed in [87]. Although transport of ROL into cells via the STRA6 transporter does occur, the predominant method of retinoid entry into embryo cells is membrane diffusion, and STRA6 function is not required for most aspects of embryo development. Mouse mutants with near complete elimination of RA signaling die at early organogenesis stages of development [88,89,90]. In contrast, *Stra6* mutant mice are viable and fertile and have very mild phenotypes. The relatively mild phenotypes of *Stra6* mutants demonstrate that STRA6 is required for maintaining vitamin A homeostasis primarily in the eye in mice [91]. In humans, mutation of *Stra6* is associated with variable phenotypes, including more severe abnormalities such as pulmonary hypoplasia-diaphragmatic hernia anophthalmia-cardiac defect (PDAC) syndrome, Matthew-Wood syndrome, and syndromic microphthalimia 9 [92,93,94]. The variable defects observed in humans with *Stra6* mutation are clearly deleterious, but they are less catastrophic than the post-implantation lethality that occurs from complete loss of RA signaling [88,89,90]. Thus, ROL can enter embryo cells either by transport or diffusion, but transport through STRA6 is essential only in a limited range of tissues, namely, eye, heart, diaphragm, and lung. Once inside the cell, the highly hydrophobic ROL is sequestered from the aqueous cytosol either by localization to membranes, or by binding to cellular retinol binding proteins [39,95]. RBP1 may play a role in trafficking retinoid ligands to membrane compartments within cells [96,97].

## 6. Reciprocal Interconversion of ROL and RAL by Membrane-Bound RDH10 and DHRS3 

Inside the cells of a developing embryo ROL can be interconverted to other retinoids, either for storage as RE, or to generate active RA for signaling. Conversion of ROL to the storage form RE, is mediated by the action of enzyme lecithin:retinol acyltransferase (LRAT) ([98] reviewed in [99]). Conversion of ROL to the active signaling retinoid, RA, occurs in two sequential oxidation steps. First ROL is oxidized to the intermediate RAL, and RAL is then subsequently oxidized to generate RA. In developing embryos the first step is rate limiting and is mediated by NAD/NADP dependent retinol dehydrogenases [100]. The primary enzyme responsible for oxidation of ROL to RAL in developing embryos is retinol dehydrogenase 10 (RDH10). The physiological relevance of RDH10 in regulating RA production during embryogenesis was identified by characterization of a mouse mutant with developmental abnormalities reminiscent of vitamin A deficiency [101]. RDH10 enzymatic activity had been previously characterized in the context of the vision cycle in adult animals, but its essential biological function in generating RA during embryonic development was not initially appreciated [102]. Analysis of enzyme kinetics of human RHD10 clarified that the enzyme uses NAD^+^ as a cofactor and free ROL as a substrate [103].

Characterization of a number of different mouse mutants revealed that loss of RHD10 function disrupts RA production and, consequently, causes multiple developmental malformations [89,101,104,105]. The embryonic defects of *Rdh10* mutants can be largely rescued by dietary supplementation with the metabolic intermediate RAL or the product RA, demonstrating that the essential function of RDH10 during embryogenesis is the production of RAL to generate RA [89,101,105].

The role RDH10 in embryonic metabolism of vitamin A is not unique to mammals. RDH10 homologs in frog and fish embryos function similarly to the RDH10 enzyme in mice. In frog embryos, overexpression of *rdh10* in the presences of ROL substrate results in a phenotype similar to treatment with excess RA, while morpholino knockdown of *rdh10* results in a moderate RA deficiency phenotype with eyes, head, and axis extension defects [106]. In zebrafish, morpholino knockdown of *Rdh10a* results in a mild RA deficiency phenotype [107]. It is possible that the loss of function phenotypes are relatively mild in these species because other SDR enzymes, such as the closely-related SDR165c *(*also known as *RDHE2)*, function redundantly with Rdh10 [108]. Alternatively, it is possible that frog and fish are less dependent on RDH10 than mice because the egg embryos have more RAL available in their lipid stores than is available to mammalian embryos and they may therefore be less dependent upon oxidation of ROL [109,110]. In humans, there has been no report of mutation of *RDH10* associated with developmental malformation or birth defect syndrome, likely because RDH10 function is essential for mammalian embryo survival, and loss of function is lethal prior to birth.

The RDH10-mediated oxidation of ROL to RAL is a nodal point in regulation of RA production during embryogenesis. *Rdh10* gene expression is regulated by feedback from RA signaling in mice and frogs [90,106]. However, in chick, *Rdh10* gene expression is not responsive to ectopic RA [111], demonstrating that RA feedback regulation on *Rdh10* does not occur in all species. 

RDH enzymes are members of the short chain dehydrogenase/reductases (SDR) superfamily of proteins [112]. The protein superfamily is very large and evolutionarily deep, and a systematic nomenclature system has been established to organize the related genes within, and between, species (Table 1) [113]. RDH10 is a member of the SDR16C family of SDR enzymes [113,114]. Like other SDR family members, RDH10 is a membrane-bound enzyme. It contains a conserved single-pass helical signal-anchor sequence at the N-terminus of the protein, and an additional C-terminal hydrophobic domain. Both domains contribute to localization in membrane compartments within cells [115,116]. The membrane localization of RDH10 may enable the enzyme to access free ROL (associated with a lipid membrane) as substrate, as opposed to ROL bound to RBP1 as it would be in the aqueous cytoplasm [39,117,118,119,120].

Although it is clearly important, RDH10 cannot be the only enzyme contributing to production of RAL during embryogenesis. RA production is not completely eliminated in *Rdh10* null mouse mutants [89,101]. Embryos can also generate RAL from β-carotene by the action of the enzyme BCO1 (also known as CMO1, Bcmo1) [44]. Additionally, there may be SDR enzymes other than RDH10 that oxidize ROL to RAL within in an embryo. A potential candidate enzyme is SDR16C5 (also known as RDHE2), which oxidizes ROL to RAL and is closely related to RDH10 [108,121]. SDR9C family members *Rdh1* or *Rdh16*, or SDR7C family member *Rdh11* are unlikely to be responsible for embryonic RA production as mutants of these genes do not have embryonic phenotypes [122,123]. 

A second class of enzymes, the ADH family of cytosolic alcohol dehydrogenases, is biochemically capable of oxidizing ROL to RAL in vitro. Owing to this biochemical activity, ADH enzymes have been cited as being responsible for RA production within embryos. However, ADH family members do not contribute to embryonic vitamin A metabolism under normal conditions in vivo. Deletion of all five ADH family members does not disrupt RA production or embryonic development [124]. Instead the ADH family enzymes play a role in retinoid homeostasis under conditions of deficiency or excess in adult animals. Adult mice with a mutation of *Adh1* are hypersensitive to vitamin A excess [125,126]. Mice with *Adh7* (formerly *Adh4)* mutation are hypersensitive to vitamin A deficiency [125,127], and mice with a mutation of *Adh5* (formerly *Adh3*) are hypersensitive to both excess and deficiency of vitamin A [128]. ADH enzymes are cytosolic, and within the cytosol ROL is bound by RBP1. ADHs can oxidize free ROL in vitro, but cannot oxidize ROL bound to RBP1 [129,130]. The likely reason that ADH enzymes do not contribute to physiological ROL metabolism under normal conditions is that there is no unbound ROL available as substrate within the cytoplasm.

In addition to being the rate-limiting step in production of RA [100,131], the conversion of ROL to RAL is also a reversible reaction. In developing embryos, the reverse of RDH10-mediated ROL oxidation, that is, the reduction of RAL to ROL, is mediated by dehydrogenase/reductase (SDR family) member 3 (DHRS3). Like RDH10, the enzymatic function of DHRS3 was originally analyzed in the context of enzymes involved in vision in the adult eye [132]. The importance of DHRS3 in retinoid metabolism during embryogenesis was identified initially on the basis of gene expression analysis of zebrafish embryos following treatment with excess RA or inhibition of RA synthesis or signaling [133]. Expression of *dhrs3* was upregulated when RA was excessive and downregulated when RA synthesis or signaling was blocked, demonstrating that *dhrs3* gene transcription was responsive to feedback regulation by RA. The critical role of DHRS3 in regulating metabolism of RA during embryonic development is conserved in other vertebrate species. In mice, mutation of *Dhrs3* leads to depletion of stored retinoid precursors and build-up of excess RA in embryos, causing multiple malformations and late-gestation lethality [134,135]. In frogs, knock-down of *Dhrs3* causes embryonic defects similar to treatment with excess RA [136].

RDH10 and DHRS3 are each oxidoreductases, redox enzymes capable of catalyzing oxidation or reduction depending on the availability of substrate and cofactor. Cofactors for such enzymes exist in different redox states, for example NAD^+^ (oxidized) versus NADH (reduced), or NADP^+^ versus NADPH. For oxidoreductases that can utilize the two different forms of the same cofactor, the direction of the redox reaction is defined by the availability of substrate and the form of cofactor available. Enzyme kinetic analysis demonstrates that RHD10 can catalyze either oxidation of ROL or reduction of RAL in vitro [103]. The preferred cofactor for RDH10 is NAD^+^ [103], and the preferred cofactor for DHRS3 is NADPH [132]. In adult rat liver cells, the amount of NAD^+^ exceeds NADH by ~1000-fold, and the amount of NADPH exceeds NADP+ by ~100-fold [137]. The high ratio of NAD^+^/NADH in vivo ensures that RDH10 functions primarily as an oxidase in living tissues. Conversely, the high ratio of NADPH/NADP^+^ ensures that DHRS3 functions primarily as a reductase. 

RDH10 and DHRS3 work coordinately together to regulate interconversion of ROL and RAL during embryo development. The two enzymes are closely related proteins (Table 1). Human DHRS3 shares 54% sequence identity with human RDH10 at the amino acid level. Both are integral membrane proteins [115,116,138]. Importantly, the enzymes reciprocally activate each other when the two proteins are present together on the same membrane [135]. Thus, the reversible first step in metabolism of vitamin A occurs not in the cytosol but in close association with a membrane compartment, mediated by two membrane-bound redox enzymes that functionally interact.

## 7. RAL Oxidized to RA by ALDH1A Enzymes

In contrast to the reversible ROL-RAL interconversion first step of vitamin A metabolism, oxidation of RAL to the active signaling molecule RA is an irreversible reaction. Oxidation of RAL to RA is mediated by the ALDH1A family of enzymes [139,140]. Three ALDH1A family members; ALDH1A1, ALDH1A2, and ALDH1A3 (also known as RALDH1, RALDH2, and RALDH3), are expressed and active during embryonic development. *Aldh1a2* is expressed earliest, from the neurulation stage onward [141], and the gene product ALDH1A2 is responsible for a majority of RAL-to-RA oxidation within a developing embryo. Targeted knock out of *Aldh1a2* in mice results in near complete elimination of RA production and embryo lethality by mid-gestation [88]. *Aldh1a1* and *Aldh1a3* are expressed later, after the onset of facial morphogenesis. ALDH1A3 is required for morphogenesis of the eyes and nasal structures [142]. ALDH1A1 contributes for formation of the dorsal retina [143,144]. RAL, like ROL, is hydrophobic. The question of how hydrophobic RAL reaches the ALDH1 enzymes remains to be determined.

## 8. Fate of RA: Nuclear Transport, Catabolism, or Movement to Other Cells

Once RA is generated it can enter the nucleus to interact with RARs, it can be catabolized, or it can move to neighboring cells. There are two cellular retinoic acid binding proteins (CRABP). CRABP2 shuttles RA into the nucleus and delivers it directly to RARs [145,146,147]. CRABP1 may sequester RA from the aqueous cytoplasm, or shuttle it to catabolizing enzymes [148]. Crabp proteins are essential for hindbrain patterning in zebrafish [149]. However, in mice, mutation of *Crabp2* causes only minor limb abnormalities that are mild relative to defects in RA production [150]. Mutation of *Crabp1* in mouse produces no detectable embryonic phenotype [151,152]. Thus, these binding proteins may be dispensable, or other factors may compensate for their loss, during mammalian embryogenesis. 

Maintaining the appropriate level and distribution of RA within developing embryonic tissues depends upon controlled elimination of RA as well as RA production. Active RA is catabolized to inactive metabolites by oxidation by CYP26 family enzymes, which are members of the cytochrome P450 superfamily of proteins [153] (reviewed in [154,155]). Two CYP26 enzymes, CYP26A1 and CYP26B1 are essential for spatially restricted clearance of RA in developing vertebrate embryos. Mutation of *Cyp26a1* or *Cyp26b1* in mice results in defects similar to treatment with excess RA [156,157]. A third paralog, CYP26C1, is not essential on its own, but functions redundantly with CYP26A1 [158]. 

RA produced in one cell can result in signaling activity in neighboring cells, thereby functioning in a paracrine manner (reviewed in [159]). The mechanism of RA movement has not been demonstrated experimentally, but could result from diffusion or transport. The ability of RA to diffuse would be consistent with a function as a morphogen, that is, action on cells at a distance from the source of production. RA could form a gradient by diffusion from a source tissue with high concentration of RA toward a sink tissue with vanishingly low concentration of RA. Source tissues could be those that express high levels of RA producing enzymes, and sink tissues could be those that express enzymes of RA catabolism. Gradients of RA have been detected by chemical analysis in chick [77,78], by direct molecular sensors [79], and by using reporters of RA signaling activity in zebrafish [160]. Computational modeling of RA gradients has identified features that make them robust (reviewed in [161]). 

## 9. Tissue-Specific Gene Expression and Feedback Regulation

Genes involved in production of RA, including *Rdh10*, *Dhrs3*, *Aldh1a1*, *Aldh1a2*, *Aldh1a3*, are expressed in, or near, cells with active RA signaling [54,101,162,163,164,165,166,167]. Conversely, genes involved in RA catabolism are expressed in regions of the embryo where RA signaling is absent [167,168,169]. Many of the vitamin A metabolic, or catabolic genes are controlled by positive and negative feedback from RA signaling (reviewed in [170]). Genes that are transcriptionally regulated by feedback from RA include those involved in vitamin A metabolism and RA production [104,106,133,134,164]. Genes encoding RARs are also sensitive to feedback control [171], as are genes encoding the RA catabolizing CYP26 enzymes [172].

## 10. Development of Many Embryo Systems Depends upon Genes and Proteins Involved in Retinoid Transport, Metabolism, Signaling, and Catabolism

A complete description of all aspects of embryogenesis that are regulated by RA signaling is beyond the scope of this review. Table 2 provides a partial list of embryonic systems that are disrupted when genes required for retinoid metabolism, catabolism, or signaling are mutated or experimentally knocked down.

## 11. Summary

RA, the active metabolite of vitamin A, is a signaling molecule essential for many aspects of embryonic development. The amount and distribution of RA signaling is precisely regulated in specific tissues and specific domains of a developing embryo. Tight control of RA signaling is achieved by regulated expression of genes encoding proteins that mediate vitamin A metabolism, retinoid transport, nuclear signaling, and RA catabolism. Recent studies have highlighted the importance of the reversible first step of vitamin A metabolism, the inter-conversion of ROL and RAL mediated by RDH10 and DHRS3, two related membrane-bound SDR proteins that activate each other functionally. The mechanisms and functions of vitamin A metabolism and RA signaling have been revealed by nutritional deficiency studies, exposure to ectopic RA, and by the phenotypes of mutants in model experimental organisms. Mutations that disrupt retinoid metabolism or RA signaling cause a wide range of defects impacting nearly all parts of an embryo. Although the list of known functions of RA signaling is extensive, future studies will undoubtedly identify additional, as yet unknown, roles for RA signaling in embryonic development. Application of new large-scale sequencing technologies will likely identify new targets of direct RA transcriptional regulation. Given the exciting new observations of non-genomic functions of RA in vitro, future exploration of these novel RA functions in vivo promises to reveal interesting roles for vitamin A and its derivatives during embryonic development.

## Figures and Tables

**Table 1:** Nomeclature of SDR16C enzymes RDH10 and DHRS3 from model organisms. For complete list of SDR enzymes see: www.sdr-enzymes.org.

Common Name	SDR Family	SDR16C Family Member ID	Organism	Other Names
RDH10	SDR16C	4	*Homo sapiens*	
	SDR16C	10	*Mus musculus*	
	SDR16C	15	*Rattus norvegicus*	
	SDR16C	81	*Xenopus laevis*	*rdh10b*
	SDR16C	82	*Xenopus laevis*	*rdh10a*
	SDR16C	36	*Danio rerio*	*rdh10-a*
	SDR16C	37	*Danio rerio*	*rdh10-b*
DHRS3	SDR16C	1	*Homo sapiens*	*RDH17*, *Rsdr1*, *SDR1*, *retSDR1*
	SDR16C	9	*Mus musculus*	*Rsdr1*, *retSDR1*
	SDR16C	16	*Rattus norvegicus*	*rCG_31196*
	SDR16C	83	*Xenopus laevis*	
	SDR16C	39	*Danio rerio*	*dhrs3b*
	SDR16C	40	*Danio rerio*	*dhrs3a*
	SDR16C	129	*Gallus gallus*	

**Table 2:** Mutations or morpholino knock-down of genes involved in vitamin A metabolism and RA signaling impact development of many embryonic systems.

System	Review	Transport, and Binding Proteins	Metabolism of ROL to RA	Canonical Signaling	RA Elimination
		RBP4, STRA6, CRABP2	RHD10, DHRS3, BCO1, ALDH1A1, ALDH1A2, ALDH1A3	RARα, RARβ, RARγ	CYP26A1, CYP26B1, CYP26C1
Anterior-posterior patterning: Brain/hindbrain	[173]	[149]	[89,133,174,175,176]	[177,178]	[156,157,158,160]
Anterior-posterior patterning: axial skeleton				[179,180]	[156,157]
Limb			[89,101,175,181,182,183,184]	[180,185,186]	
Mid-face, nasal cavity, and Palate	[187]	[89,101,188] * [44] ** [98]	[89,101,134,135,142]	[180]	[189]
Ear	[190,191,192]		[89,90,101]	[180]	
Eye		[92,93,94,193] * [44] ** [98]	[89,101,135,142]	[180]	
Digestive system			[89]	[185,186]	
Liver			[89,101,194]	[195]	
Lung/trachea			[89,101,194]		
Pancreas			[194,196]		
Heart			[89,90,107,134,174,197]	[171,185,186]	
Spleen			[198]		
Somite formation			[199]	[200]	
Posterior axis extension, tail formation				[201]	[156,157]
Kidney/Urogenital			[89]	[185,186,202,203]	[156,157]
Germ cells	[204]		[205]		[206,207,208]
Blood cells			[209]		

* Compound mutation with *Bco1−/−*; *Lrat−/−* with vitamin A deficient diet; ** Compound mutant *Rbp4−/−*; *Lrat−/−* with vitamin A deficient diet.
